# Increased prevalence of clonal hematopoiesis of indeterminate potential amongst people living with HIV

**DOI:** 10.1038/s41598-021-04308-2

**Published:** 2022-01-12

**Authors:** Alexander G. Bick, Konstantin Popadin, Christian W. Thorball, Md Mesbah Uddin, Markella V. Zanni, Bing Yu, Matthias Cavassini, Andri Rauch, Philip Tarr, Patrick Schmid, Enos Bernasconi, Huldrych F. Günthard, Peter Libby, Eric Boerwinkle, Paul J. McLaren, Christie M. Ballantyne, Steven Grinspoon, Pradeep Natarajan, Jacques Fellay, the Swiss HIV Cohort Study, I. Abela, K. Aebi-Popp, A. Anagnostopoulos, M. Battegay, E. Bernasconi, D. L. Braun, H. C. Bucher, A. Calmy, M. Cavassini, A. Ciuffi, G. Dollenmaier, M. Egger, L. Elzi, J. Fehr, J. Fellay, H. Furrer, C. A. Fux, H. F. Günthard, A. Hachfeld, D. Haerry, B. Hasse, H. H. Hirsch, M. Hoffmann, I. Hösli, M. Huber, C. R. Kahlert, L. Kaiser, O. Keiser, T. Klimkait, R. D. Kouyos, H. Kovari, K. Kusejko, G. Martinetti, B. Martinez de Tejada, C. Marzolini, K. J. Metzner, N. Müller, J. Nemeth, D. Nicca, P. Paioni, G. Pantaleo, M. Perreau, A. Rauch, P. Schmid, R. Speck, M. Stöckle, P. Tarr, A. Trkola, G. Wandeler, S. Yerly

**Affiliations:** 1 Division of Genetic Medicine, Department of Medicine, Vanderbilt University School of Medicine, Nashville, TN USA; 2 School of Life Sciences, École Polytechnique Fédérale de Lausanne, Station 19, 1015 Lausanne, Switzerland; 3 Swiss Institute of Bioinformatics, Lausanne, Switzerland; 4 Precision Medicine Unit, Lausanne University Hospital and University of Lausanne, Lausanne, Switzerland; 5 Broad Institute of MIT and Harvard, Cambridge, MA USA; 6 Metabolism Unit, Massachusetts General Hospital and Harvard Medical School, Boston, MA USA; 7 Human Genetics Center, Baylor College of Medicine, University of Texas Health Science Center, Houston, TX USA; 8 Service of Infectious Diseases, Lausanne University Hospital and University of Lausanne, Lausanne, Switzerland; 9 Department of Infectious Diseases, Bern University Hospital, University of Bern, Bern, Switzerland; 10 Department of Infectious Diseases and Hospital Epidemiology, University Hospital Basel, University of Basel, Basel, Switzerland; 11 Division of Infectious Diseases and Hospital Epidemiology, Cantonal Hospital St.Gallen, St.Gallen, Switzerland; 12 Division of Infectious Diseases, Regional Hospital of Lugano, Lugano, Switzerland; 13 Department of Infectious Diseases and Hospital Epidemiology, University Hospital Zurich, Zurich, Switzerland; 14 Institute of Medical Virology, University of Zurich, Zurich, Switzerland; 15 Division of Cardiovascular Medicine, Department of Medicine, Brigham and Women’s Hospital, Harvard Medical School, Boston, MA USA; 16 JC Wilt Infectious Diseases Research Centre, National Microbiology Laboratory, Public Health Agency of Canada, Winnipeg, Canada; 17 Department of Medical Microbiology and Infectious Diseases, University of Manitoba, Winnipeg, Canada; 18 Department of Medicine, Baylor College of Medicine, Houston, TX USA; 19 Cardiovascular Research Center, Massachusetts General Hospital, 185 Cambridge Street, CPZN 3.184, Boston, MA 02114 USA; 20 Basel Institute for Clinical Epidemiology and Biostatistics, University Hospital Basel, University of Basel, Basel, Switzerland; 21 Institute of Microbiology, University Hospital Lausanne, University of Lausanne, Lausanne, Switzerland; 22 Centre for Laboratory Medicine, St. Gallen, Canton St. Gallen, Switzerland; 23 Institute of Social and Preventive Medicine, University of Bern, Bern, Switzerland; 24 Clinic for Infectious Diseases and Hospital Hygiene, Kantonsspital Aarau, Aarau, Switzerland; 25 Deputy of the Patient Organization “Positive Council”, Zurich, Switzerland; 26 Division Infection Diagnostics, Department Biomedicine - Petersplatz, University of Basel, Basel, Switzerland; 27 Clinic for Obstetrics, University Hospital Basel, University of Basel, Basel, Switzerland; 28 Childrens Hospital of Eastern Switzerland, St. Gallen, Switzerland; 29 Division of Infectious Diseases and Laboratory of Virology, University Hospital Geneva, University of Geneva, Geneva, Switzerland; 30 Institute of Global Health, University of Geneva, Geneva, Switzerland; 31 Data Centre Swiss HIV Cohort Study, University Zurich, Zurich, Switzerland; 32 Cantonal Institute of Microbiology, Bellinzona, Switzerland; 33 Department of Obstetrics and Gynecology, University Hospital Geneva, University of Geneva, Geneva, Switzerland; 34 University Children’s Hospital, University of Zurich, Zurich, Switzerland; 35 Division of Immunology and Allergy, University Hospital Lausanne, University of Lausanne, Lausanne, Switzerland; 36 Laboratory of Virology, University Hospital Geneva, University of Geneva, Geneva, Switzerland

**Keywords:** Immunology, Infectious diseases, HIV infections, Population genetics, Genomics

## Abstract

People living with human immunodeficiency virus (PLWH) have significantly increased risk for cardiovascular disease in part due to inflammation and immune dysregulation. Clonal hematopoiesis of indeterminate potential (CHIP), the age-related acquisition and expansion of hematopoietic stem cells due to leukemogenic driver mutations, increases risk for both hematologic malignancy and coronary artery disease (CAD). Since increased inflammation is hypothesized to be both a cause and consequence of CHIP, we hypothesized that PLWH have a greater prevalence of CHIP. We searched for CHIP in multi-ethnic cases from the Swiss HIV Cohort Study (SHCS, n = 600) and controls from the Atherosclerosis Risk in the Communities study (ARIC, n = 8111) from blood DNA-derived exome sequences. We observed that HIV is associated with a twofold increase in CHIP prevalence, both in the whole study population and in a subset of 230 cases and 1002 matched controls selected by propensity matching to control for demographic imbalances (SHCS 7%, ARIC 3%, *p* = 0.005). We also observed that *ASXL1* is the most commonly mutated CHIP-associated gene in PLWH. Our results suggest that CHIP may contribute to the excess cardiovascular risk observed in PLWH.

## Introduction

As current treatments have rendered human immunodeficiency virus (HIV) a chronic condition, coronary artery disease has emerged as a major source of morbidity in people living with human immunodeficiency virus (PLWH). Inflammation and immune dysregulation likely accelerate CAD risk among PLWH^1^. Recently, ‘clonal hematopoiesis of indeterminate potential’ (CHIP), the age-related acquisition and expansion of leukemogenic mutations (primarily in *DNMT3A*, *TET2*, *ASXL1*, *JAK2*) in white blood cells, was found to increase risk for both hematologic malignancy^2,3^ and CAD^4,5^ among asymptomatic individuals in the general population. The proatherogenic mechanisms for CHIP included heightened inflammation^4,6^. Given converging proposed mechanisms promoting CAD risk and increased hematologic malignancy risk among PLWH, we tested the hypothesis that HIV-infected individuals have heightened prevalence of CHIP.

## Methods

We identified CHIP in a multi-ethnic sample of 600 PLWH from the Swiss HIV Cohort Study (SHCS), aged 21–83. The SHCS is a multicenter, prospective observational study for interdisciplinary HIV research^7^. Established in 1988, the SHCS currently comprises more than 20,000 PLWH with median 51 years of age. Samples of 600 patients, used for exome sequencing, were chosen randomly in terms of gender (genetic sex, gender at birth), age, category of transmission, as well as HIV management and control^8^.

We utilized a set of 8111 individuals with available exome sequences from the Atherosclerotic Risk in the Community study (ARIC), aged 45–84 years, as population controls^9^. The ARIC study is a prospective longitudinal investigation of the development of atherosclerosis and its clinical sequelae which enrolled 15,792 individuals aged 45 to 64 years at baseline^10^. At study enrollment (1987–1989), the participants were selected by probability sampling from four United States communities: Forsyth County, North Carolina; Jackson, Mississippi; the northwestern suburbs of Minneapolis, Minnesota; and Washington County, Maryland.

Exome capture kits used in the compared cohorts were different (SHCS: xGen Exome Research Panel v 1.0, Sureselect All Exon V5 and TruSeq DNA Exome, ARIC: HGSC VCRome 2.1 design (42 Mb, NimbleGen)), which is the major limitation of the study. To minimise these differences we performed a set of statistical analysis to normalize the coverage among cohorts (see below the adjusting for the depth of sequencing and table S4).

CHIP was called in both exome sequenced cohorts using an identical and previously described pipeline^4,11^. Briefly, short read sequence data were aligned to the hg19 reference genome using the BWA-mem algorithm and processed with the Genome Analysis Toolkit MuTect2 tool to detect somatic variants^12^. To identify individuals with CHIP, we used a pre-specified list of variants in 74 genes known to be recurrent drivers of myeloid malignancies (Table S3) and variant filtration process (> = 20 reads in total, >  = 3 Alt reads including one on a forward and one on a reverse strand, VAF limit > 2%) which filter in the biologically relevant cases^13^.

As CHIP prevalence depends strongly on age, we performed a 1:5 case/control propensity matching on age, sex and self-reported ethnicity using nearest neighbor matching^14^ as implemented by the MatchIt package version 3.0.2 in R. Next, we used univariate Fisher’s exact test and multivariable logistic regression to test the association between HIV status and CHIP prevalence. Multivariable models were adjusted for age, sex, self-reported ethnicity, and smoking status.

To take into account potential difference in the depth of sequencing of the CHIP-associated genes between the matched cohorts, we used a backward stepwise multiple logistic model, describing CHIP status (0/1) as a function of cohort (0/1) and coverage of the four most common CHIP-associated genes (*DNMT3A*, *TET2*, *ASXL1* and *JAK2*). Analyses were performed in R version 3.6. A threshold of *p* < 0.05 was considered statistically significant.

The Swiss HIV Cohort Study was approved by the local ethical committees of the participating centres: Ethikkommission beider Basel ("Die Ethikkommission beider Basel hat die Dokumente zur Studie zustimmend zur Kenntnis genommen und genehmigt."); Kantonale Ethikkommission Bern (21/88); Comité départemental d'éthique des spécialités médicales et de médecine communautaire et de premier recours, Hôpitaux Universitaires de Genève (01–142); Commission cantonale d'éthique de la recherche sur l'être humain, Canton de Vaud (131/01); Comitato etico cantonale, Repubblica e Cantone Ticino (CE 813); Ethikkommission des Kantons St. Gallen (EKSG 12/003); Kantonale Ethikkommission Zürich (KEK-ZH-NR: EK-793), and written informed consent was obtained from all participants. Secondary analysis of the SHCS data in this manuscript was covered by the original approvals mentioned above, and secondary analysis of ARIC was approved by the Mass General Brigham Institutional Review Board.

All methods were performed in accordance with the relevant guidelines and regulations.

## Results

**Figure 1:**
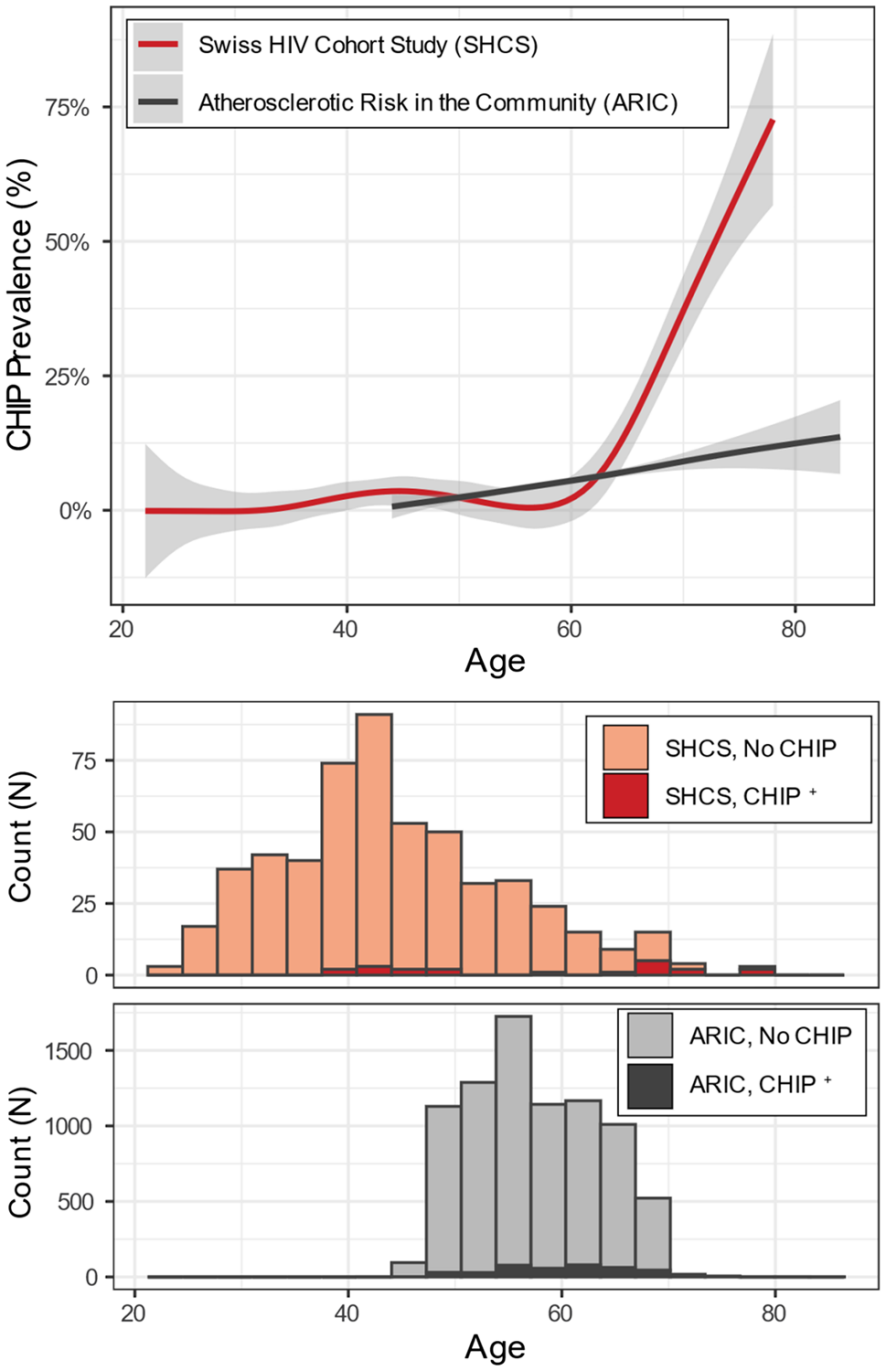
CHIP prevalence in Swiss HIV Cohort Study and Atherosclerotic Risk in the Community Study. Upper panel: fraction of cohort observed to have CHIP over time fit with a general additive model spline. 95% confidence interval displayed as shaded area. Lower panel: Count of number of individuals with and without CHIP binned by age of time of blood sampling across the entire sequenced cohort. Complete datasets (SHSC N = 600, ARIC N = 8111).

First, we compared the prevalence of CHIP across the entire SHCS PLWH cohort (N = 600) and ARIC cohort (N = 8111) (Fig. 1). SHCS PLWH and ARIC participants had mean (SD) age 44 (11) and 57 (6) years (*p* = 1.8 × 10^–167^), were 25% and 56% female (*p* = 1.9 × 10^−46^), and were 95% and 74% of European ancestry (*p* = 5.2 × 10^−36^) respectively. With adjustment for age, age^2^, sex and self-reported ethnicity, we observed a significant association between HIV case status and CHIP (OR: 1.77, 95% CI: 1.33–2.21, *p* = 0.02).

Second, given the overall demographic imbalances, to confirm an excess of CHIP amongst SHCS under all else equal, we used a propensity matching strategy to match the two cohorts by age, gender, self-reported ethnicity and smoking status (ever-smoker or not). Propensity matching analyses yielded a set of 230 (out of 600) PLWH cases and 1002 (out of 8111) ARIC population controls. Neither age nor sex differed significantly between the matched cohorts (Table 1) and the standardized mean difference across age, sex and self-reported ethnicity were all less than 0.1 indicative of adequate matching. In this subset, CHIP was detected in 7% of exomes from PLWH, but only 3% of the controls (Table 1, univariate *p* = 0.005; multivariable *p* = 0.004). Of note, the statistical association strengthened despite a significantly decreased sample size, demonstrating the robustness of our matching approach.

**Table 1:** Demographics and CHIP association in matched samples.

n	HIV + Individuals (SHCS)	Population Controls (ARIC)	*p*-value
	230	1002	
Age at blood draw, mean (st. dev.)	54.2 (7.4)	55.0 (6.8)	0.12
Female, N (%)	44 (19%)	240 (24%)	0.086
Ever smoker, N (%)	143 (62%)	651 (65%)	0.408
Diabetes mellitus, N (%)	18 (8%)	80 (8%)	0.936
Black, N (%)	7 (3%)	80 (8%)	0.017
CHIP carrier, N (%)	16 (7%)	30 (3%)	0.005

*P*-value derived from Fisher's exact test for counts and t-test for continuous variables.

Third, we tested if sequencing coverage differs between the matched sub cohorts. Comparing the average coverage of the four most common CHIP genes (DNMT3A, TET2, ASXL1, JAK2) between the matched SHCS and ARIC sub cohorts we observed higher coverage in SHCS (median coverages are 66 and 47 reads per nucleotide for SHCS and ARIC correspondingly, *p* < 2 * 10^−16^ Mann–Whitney U test). Increased sequence coverage in SHCS can facilitate CHIP discovery in SHCS and to take into account the differences in the coverage we performed a multivariable logistic regression analysis with the included depth of sequencing. Inclusion into the models the total coverage of the four most common CHIP genes (*DNMT3A*, *TET2*, *ASXL1*, *JAK2*) as well as individual coverage of those genes demonstrated non-significant effect of the coverage (models 2 and 3 in table S4), confirming the robustness of our main finding—an excess of CHIP amongst PLWH (model 1 in table S4).

The limited sample size precluded inference on the association of HIV status with specific CHIP driver genes, however we observed differences in the genes most likely to carry CHIP mutations between PLWH (table S1) and population controls (table S2). The most common CHIP gene in the SHCS was *ASXL1* (13 out of 27 CHIP mutations, 48%) followed by *TET2* (8 out of 27 CHIP mutations, 30%) and *DNMT3A* (5 out of 27 CHIP mutations, 19%). Overall this distribution was inverted from the control cohort where CHIP mutations were more frequent in *DNMT3A (14 out of 28 CHIP mutations, 50%)*, followed by *TET2 (5 out of 28 CHIP mutations, 18%)* and *ASXL1 (5 out of 28 CHIP mutations, 18%)*. In total, 22 PLWH had a single CHIP mutation, while one individual had 2 mutations and one individual had 3 mutations, while in the ARIC cohort all CHIP carriers had a single CHIP mutation (tables S1 and S2). Additionally, we compared VAFs between matched ARIC and SHCS CHIP carriers (tables S1 and S2) and observed a trend of increased VAF in ARIC (28 CHIP mutations in ARIC and 14 CHIP mutations in SHCS: 12 patients among which one patient has 3 CHIP variants, *p*-value = 0.026, Mann–Whitney U test).

Within the full PLWH cohort (N = 600) we considered additional phenotypes, which might be a cause or consequence of CHIP. First, we observed a trend toward an increase in CAD among CHIP carriers (Fisher’s exact test OR: 2.99, *p* = 0.068) and increased cases of diabetes among CHIP carriers (Fisher’s exact test OR: 3.76, *p* = 0.037) (see the patient-specific information in Table S1). Second, we observed that duration of antiretroviral therapy (ART) was twice as long in CHIP carriers versus non-carriers (ART mean [st. dev.] 2675 [1850] days vs. 1322 [1454] days in carriers vs. non-carriers, respectively; *p* = 0.0004, Mann–Whitney U test). This association was directionally concordant after adjusting for patient age in multiple logistic regression (*p* = 0.066). It is important to note that although ART duration positively correlated with the total duration of HIV infection (Spearman’s rho = 0.58, *p* = 2.0 × 10^−54^), the total duration of HIV infection was not associated with CHIP (*p* = 0.452; paired Mann–Whitney U test on matched CHIP carriers and non-carriers, *p* = 0.22).

## Discussion

Here, we report that HIV infection is associated with increased prevalence of CHIP. In the present samples, we identify a more than twofold enrichment of CHIP among PLWH versus controls after careful adjustment for known factors predisposing to CHIP (age, smoking status, ethnicity, gender). Although PLWH and controls originate from different cohorts, identical bioinformatics pipelines were used for the identification of CHIP-associated variants, and the statistical matching of cohorts and multiple logistic models controlling for the gene coverage (see Methods) assure robustness of our results. Of note, a very similar twofold excess of CHIP among PLWH has been described recently in an independent study from Australia^15^. Altogether, we demonstrate that HIV infection is the second strongest factor, after age, associated with increased CHIP prevalence among PLWH.

Our finding is based on cross‐sectional design and thus we cannot infer causality. However, assuming that CHIP is highly unlikely to be a risk factor for HIV acquisition, we focus on potential mechanisms that could promote CHIP development among PLWH. HIV infection or, more generally, HIV-related factors may promote CHIP development either through an increased rate of occurrence of CHIP-associated mutations and/or increased rate of clonal expansion of these somatic variants.

An increased somatic mutational rate in PLWH up to date has been shown only for the mitochondrial genome, which is particularly sensitive to some antiretroviral therapies^16–19^. However, taking into account several potential factors such as: a mutagenic effect of the virus, DNA-replication errors associated with an increased turnover rate of hematopoietic stem cells, mutagenic effects of antiretroviral therapy and other HIV-specific confounders (such as tobacco smoke, the effect of which has been controlled in the current study) we can not rule out an increased rate of somatic mutagenesis in the nuclear genome of PLWH.

A potentially higher rate of occurrence of somatic mutations alone is unlikely to provide a comprehensive explanation of increased CHIP prevalence among PLWH. A recent study, performing accurate detection of rare (with variant allele frequency higher than or equals to 0.0003) CHIP-associated mutations, demonstrated that such CHIP variants are nearly universal in healthy individuals by the age of 50 and often stable longitudinally^20^, showing that for the majority of people, decades elapse between the acquisition of a CHIP-associated mutation and CHIP itself. Thus, an understanding of the rate of clonal expansion of initially rare CHIP-associated variants is of great importance to shed light on the excess of CHIP among PLWH. A recent model proposed that many of the CHIP-associated mutations increase cell fitness, ensuring their proliferation with age^21^. Thus, HIV infection may modify the fitness landscape of CHIP-associated mutations, accelerating their clonal expansion and thus providing a fertile substrate for CHIP development. Various HIV-related mechanisms may be responsible for this, including induced immunodeficiency, increased prevalence of tobacco smoking and other comorbid conditions, as well as chronic immune activation from antigenic stimulation. Indeed, it has been recently shown that mutations in both the most common CHIP-associated genes *DNMT3A*^22^ and *TET2*^23^ are getting selective advantage in case of chronic infection. According to our results, ART could additionally induce CHIP development, however an elucidation of the mechanisms as well as relative contribution of different HIV-specific factors to CHIP risk requires future studies.

The relationship we identify between HIV and CHIP may be a mechanistic basis of shared phenotypes. For example, recent study showed that HIV infection leads to a greater risk of myelodysplastic syndrome (MDS), a downstream consequence of CHIP and precursor to myeloid malignancy^24^. Furthermore, similar to the gene distribution in MDS, we find a greater relative prevalence of *ASXL1* mutations among PLWH compared to controls. Of note, while cigarette smoking selects for *ASXL1* clonal hematopoiesis^25^, our cohort of PLWH still had an increased prevalence of *ASXL1* mutations compared to the control cohort despite being matched for smoking status. Another shared phenotype is an increased risk for cardiovascular disease. We propose that CHIP may be one mechanism that elevates risk for CAD among PLWH and further studies are required to evaluate this hypothesis.

PLWH have accelerated biologic aging. CHIP detection may represent a new opportunity for identification of at-risk patients with particular relevance for HIV medicine. Conversely, PLWH may provide a rich source of information to understand mechanisms of clonal expansion of different CHIP-associated variants under long-term low-grade inflammation.

## Supplementary Information

Supplementary Information.

## Data Availability

CHIP-associated genetic variant callsets and associated participant level phenotype data used in this study are available to qualified investigators by application to the SHCS and ARIC.
