# Author Correction: Increased prevalence of clonal hematopoiesis of indeterminate potential amongst people living with HIV

**DOI:** 10.1038/s41598-022-16151-0

**Published:** 2022-07-08

**Authors:** Alexander G. Bick, Konstantin Popadin, Christian W. Thorball, Md Mesbah Uddin, Markella V. Zanni, Bing Yu, Matthias Cavassini, Andri Rauch, Philip Tarr, Patrick Schmid, Enos Bernasconi, Huldrych F. Günthard, Peter Libby, Eric Boerwinkle, Paul J. McLaren, Christie M. Ballantyne, Steven Grinspoon, Pradeep Natarajan, Jacques Fellay, the Swiss HIV Cohort Study, I. Abela, K. Aebi-Popp, A. Anagnostopoulos, M. Battegay, E. Bernasconi, D. L. Braun, H. C. Bucher, A. Calmy, M. Cavassini, A. Ciuffi, G. Dollenmaier, M. Egger, L. Elzi, J. Fehr, J. Fellay, H. Furrer, C. A. Fux, H. F. Günthard, A. Hachfeld, D. Haerry, B. Hasse, H. H. Hirsch, M. Hoffmann, I. Hösli, M. Huber, C. R. Kahlert, L. Kaiser, O. Keiser, T. Klimkait, R. D. Kouyos, H. Kovari, K. Kusejko, G. Martinetti, B. Martinez de Tejada, C. Marzolini, K. J. Metzner, N. Müller, J. Nemeth, D. Nicca, P. Paioni, G. Pantaleo, M. Perreau, A. Rauch, P. Schmid, R. Speck, M. Stöckle, P. Tarr, A. Trkola, G. Wandeler, S. Yerly

**Affiliations:** 1 Division of Genetic Medicine, Department of Medicine, Vanderbilt University School of Medicine, Nashville, TN USA; 2 School of Life Sciences, École Polytechnique Fédérale de Lausanne, Station 19, 1015 Lausanne, Switzerland; 3 Swiss Institute of Bioinformatics, Lausanne, Switzerland; 4 Precision Medicine Unit, Lausanne University Hospital and University of Lausanne, Lausanne, Switzerland; 5 Broad Institute of MIT and Harvard, Cambridge, MA USA; 6 Metabolism Unit, Massachusetts General Hospital and Harvard Medical School, Boston, MA USA; 7 Human Genetics Center, Baylor College of Medicine, University of Texas Health Science Center, Houston, TX USA; 8 Service of Infectious Diseases, Lausanne University Hospital and University of Lausanne, Lausanne, Switzerland; 9 Department of Infectious Diseases, Bern University Hospital, University of Bern, Bern, Switzerland; 10 Department of Infectious Diseases and Hospital Epidemiology, University Hospital Basel, University of Basel, Basel, Switzerland; 11 Division of Infectious Diseases and Hospital Epidemiology, Cantonal Hospital St.Gallen, St.Gallen, Switzerland; 12 Division of Infectious Diseases, Regional Hospital of Lugano, Lugano, Switzerland; 13 Department of Infectious Diseases and Hospital Epidemiology, University Hospital Zurich, Zurich, Switzerland; 14 Institute of Medical Virology, University of Zurich, Zurich, Switzerland; 15 Division of Cardiovascular Medicine, Department of Medicine, Brigham and Women’s Hospital, Harvard Medical School, Boston, MA USA; 16 JC Wilt Infectious Diseases Research Centre, National Microbiology Laboratory, Public Health Agency of Canada, Winnipeg, Canada; 17 Department of Medical Microbiology and Infectious Diseases, University of Manitoba, Winnipeg, Canada; 18 Department of Medicine, Baylor College of Medicine, Houston, TX USA; 19 Cardiovascular Research Center, Massachusetts General Hospital, 185 Cambridge Street, CPZN 3.184, Boston, MA 02114 USA; 20 Basel Institute for Clinical Epidemiology and Biostatistics, University Hospital Basel, University of Basel, Basel, Switzerland; 21 Institute of Microbiology, University Hospital Lausanne, University of Lausanne, Lausanne, Switzerland; 22 Centre for Laboratory Medicine, St. Gallen, Canton St. Gallen, Switzerland; 23 Institute of Social and Preventive Medicine, University of Bern, Bern, Switzerland; 24 Clinic for Infectious Diseases and Hospital Hygiene, Kantonsspital Aarau, Aarau, Switzerland; 25 Deputy of the Patient Organization “Positive Council”, Zurich, Switzerland; 26 Division Infection Diagnostics, Department Biomedicine - Petersplatz, University of Basel, Basel, Switzerland; 27 Clinic for Obstetrics, University Hospital Basel, University of Basel, Basel, Switzerland; 28 Childrens Hospital of Eastern Switzerland, St. Gallen, Switzerland; 29 Division of Infectious Diseases and Laboratory of Virology, University Hospital Geneva, University of Geneva, Geneva, Switzerland; 30 Institute of Global Health, University of Geneva, Geneva, Switzerland; 31 Data Centre Swiss HIV Cohort Study, University Zurich, Zurich, Switzerland; 32 Cantonal Institute of Microbiology, Bellinzona, Switzerland; 33 Department of Obstetrics and Gynecology, University Hospital Geneva, University of Geneva, Geneva, Switzerland; 34 University Children’s Hospital, University of Zurich, Zurich, Switzerland; 35 Division of Immunology and Allergy, University Hospital Lausanne, University of Lausanne, Lausanne, Switzerland; 36 Laboratory of Virology, University Hospital Geneva, University of Geneva, Geneva, Switzerland

Correction to: *Scientific Reports* https://doi.org/10.1038/s41598-021-04308-2, published online 12 January 2022

The Competing interests section in the original version of this Article was incomplete.

"Dr. Libby is an unpaid consultant to, or involved in clinical trials for Amgen, AstraZeneca, Baim Institute, Beren Therapeutics, Esperion, Therapeutics, Genentech, Kancera, Kowa Pharmaceuticals, Medimmune, Merck, Norvo Nordisk, Merck, Novartis, Pfizer, Sanofi-Regeneron. Dr. Libby is a member of scientific advisory board for Amgen, Corvidia Therapeutics, DalCor Pharmaceuticals, Kowa Pharmaceuticals, Olatec Therapeutics, Medimmune, Novartis, and XBiotech, Inc. Dr. Libby’s laboratory has received research funding in the last 2 years from Novartis. Dr. Libby is on the Board of Directors of XBiotech, Inc. Dr. Libby has a financial interest in Xbiotech, a company developing therapeutic human antibodies. Dr. Libby's interests were reviewed and are managed by Brigham and Women's Hospital and Partners HealthCare in accordance with their conflict of interest policies. Dr Natarajan reported grants from Amgen during the conduct of the study and grants from Boston Scientific; grants and personal fees from Apple; personal fees from Novartis and MannBlackstone Life Sciences; and other support from Vertex outside the submitted work. None of the authors other than those already mentioned share any competing interests."

now reads:

"Dr. Libby is an unpaid consultant to, or involved in clinical trials for Amgen, AstraZeneca, Baim Institute, Beren Therapeutics, Esperion, Therapeutics, Genentech, Kancera, Kowa Pharmaceuticals, Medimmune, Merck, Norvo Nordisk, Merck, Novartis, Pfizer, Sanofi-Regeneron. Dr. Libby is a member of scientific advisory board for Amgen, Corvidia Therapeutics, DalCor Pharmaceuticals, Kowa Pharmaceuticals, Olatec Therapeutics, Medimmune, Novartis, and XBiotech, Inc. Dr. Libby’s laboratory has received research funding in the last 2 years from Novartis. Dr. Libby is on the Board of Directors of XBiotech, Inc. Dr. Libby has a financial interest in Xbiotech, a company developing therapeutic human antibodies. Dr. Libby's interests were reviewed and are managed by Brigham and Women's Hospital and Partners HealthCare in accordance with their conflict of interest policies. Dr Natarajan reported investigator-initiated grants from Amgen, Apple, AstraZeneca, Boston Scientific, and Novartis, personal fees from Apple, AstraZeneca, Blackstone Life Sciences, Foresite Labs, Novartis, Roche / Genentech, and TenSixteen Bio, is a scientific advisory board member of Esperion Therapeutics, geneXwell, and TenSixteen Bio, shareholder of geneXwell and TenSixteen Bio, and spousal employment at Vertex, all unrelated to the present work. None of the authors other than those already mentioned share any competing interests."

The original Article has been corrected.

