# Supplementary material for: Increased prevalence of clonal hematopoiesis of indeterminate potential amongst people living with HIV
Source: Sci Rep. 2022 Jan 12;12:577. doi: 10.1038/s41598-021-04308-2 (PMC8755790; doi:10.1038/s41598-021-04308-2)
Supplement: Supplementary file 1 — Supplementary Information. [file 41598_2021_4308_MOESM1_ESM.pdf]

Supplementary materials to a paper “Increased Prevalence of Clonal Hematopoiesis of Indeterminate Potential Amongst People Living with HIV”

Table S1. List of CHIP carriers from the SHCS cohort.

Sample	CHIP Gene	CHIP Variant	VAF	Used In Matched SubSet	Smoke. EverSmoke	Disease. CVD	Disease. Diabetes
1	TET2	R1516X	0.129411764705882	1	0	1	0
2	ASXL1	G645Vfs*58	0.032786885245902	0	1	0	1
3	ASXL1	E635Rfs*15	0.069364161849711	1	0	1	0
4	ASXL1	G645Vfs*58	0.039473684210526	1	1	0	0
5	ASXL1	G645Vfs*58	0.041420118343195	1	0	1	0
6	ASXL1	G645Vfs*58	0.026490066225166	0	0	0	0
7	ASXL1	G645Vfs*58	0.028571428571429	0	1	1	1
8	ASXL1	G645Vfs*58	0.068571428571429	0	0	0	0
9	ASXL1	G645Vfs*58	0.05421686746988	0	1	0	1
10	TET2	Q764Pfs*5	0.056179775280899	1	1	0	0
11	ASXL1	G645Vfs*58	0.040201005025126	0	1	0	0
12	DNMT3A	N612Lfs*34	0.04	0	0	0	0
13	DNMT3A	I495Sfs*156	0.115384615384615	1	1	0	0
14	DNMT3A	F909Lfs*13	0.029761904761905	1	0	0	1
15	DNMT3A	W795X	0.456521739130435	0	1	0	0
16	ASXL1,<br>TET2,<br>TET2	E1400Dfs*22,<br>S1290X,<br>L1646Rfs*49	0.117647058823529,<br>0.294117647058824,<br>0.364285714285714	1	1	0	0
17	TET2	G429X	0.02247191011236	0	0	0	0
18	BCOR	E902X	0.051724137931035	0	0	0	0
19	ASXL1	Y1441Mfs*9	0.03125	1	1	0	0
20	DNMT3A	W795Cfs*13	0.051724137931035	1	1	0	0
21	TET2	G563X	0.03125	1	0	0	0
22	TET2	R550X	0.028301886792453	1	1	0	0
23	ASXL1, TET2	G645Vfs*58,<br>S354X	0.0381679389312977,<br>0.2	0	1	0	0
24	ASXL1	G645Vfs*58	0.03448275862069	0	1	0	0

Table S2. List of CHIP carriers from the matched ARIC cohort.

Sample	CHIP Gene	CHIP Variant	VAF	Used In Matched Subset	Smoke. EverSmoke	Disease. CVD	Disease. Diabetes
1	DNMT3A	W795L	0.104	1	1	0	0
2	DNMT3A	R749C	0.078	1	0	0	0
3	TET2	I274fs	0.064	1	0	0	0
4	DNMT3A	R882H	0.254	1	1	0	0
5	ASXL1	G642fs	0.207	1	0	0	0
6	DNMT3A	W860X	0.098	1	0	0	0
7	ASXL1	Q529X	0.153	1	1	0	0
8	DNMT3A	W860R	0.099	1	1	0	0
9	DNMT3A	R882H	0.178	1	1	0	0
10	DNMT3A	L653F	0.258	1	1	0	0
11	DNMT3A	exon12:c.1481-1G>A	0.123	1	0	0	0
12	GNB1	K57E	0.331	1	0	0	0
13	DNMT3A	exon13:c.1717+1G>A	0.121	1	1	0	0
14	DNMT3A	I705T	0.051	1	1	0	0
15	DNMT3A	R882H	0.08	1	1	0	0
16	DNMT3A	W440X	0.082	1	1	0	0
17	TET2	S835X	0.065	1	1	1	1
18	TP53	C238Y	0.086	1	1	0	0
19	ASXL1	Y974_C975delinsX	0.143	1	1	0	0
20	ASXL1	K1303X	0.145	1	0	0	0
21	DNMT3A	exon13:c.1717+1G>A	0.097	1	0	0	0
22	TET2	S75fs	0.1	1	1	0	0
23	TET2	Q803X	0.064	1	0	0	0
24	DNMT3A	R635Q	0.352	1	0	0	0
25	ASXL1	R693X	0.177	1	1	0	0
26	BCOR	L1284fs	0.058	1	1	0	0
27	TET2	L1032fs	0.092	1	1	0	0
28	SF3B1	K700E	0.057	1	0	0	0

Table S3. List of 74 CHIP genes and variants, used to refer to the CHIP carriers.

Gene	ReportedMut	Accession
ASXL1	Frameshift/nonsense/splice-site in exon 11-12	NM_015338
ASXL2	Frameshift/nonsense/splice-site in exon 11-12	NM_018263
BCOR	Frameshift/nonsense/splice-site	NM_001123385
BCORL1	Frameshift/nonsense/splice-site	NM_021946
BRAF	G464E, G464V, G466E, G466V, G469R, G469E, G469A, G469V, V471F, V472S, L485W, N581S, I582M, I592M, I592V, D594N, D594G, D594V, D594E, F595L, F595S, G596R, L597V, L597S, L597Q, L597R, A598V, V600M, V600L, V600K, V600R, V600E, V600A, V600G, V600D, K601E, K601N, R603*, W604R, W604G, S605G, S605F, S605N, G606E, G606A, G606V, H608R, H608L, G615R, S616P, S616F, L618S, L618W	NM_004333
BRCC3	Frameshift/nonsense/splice-site	NM_024332
CBL	RING finger missense p.381-421	NM_005188
CBLB	RING finger missense p.372-412	NM_170662
CEBPA	Frameshift/nonsense/splice-site	NM_004364
CREBBP	Frameshift/nonsense/splice-site, D1435E, R1446L, R1446H, R1446C, Y1450C, P1476R, Y1482H, H1487Y, W1502C, Y1503D, Y1503H, Y1503F, S1680del	NM_004380
CSF1R	L301F, L301S, Y969C, Y969N, Y969F, Y969H, Y969D	NM_005211
CSF3R	T615A, T618I, truncating c.741-791	NM_000760
CTCF	Frameshift/nonsense, R377C, R377H, P378A, P378L	NM_006565
CUX1	Frameshift/nonsense	NM_181552
DNMT3A	Frameshift/nonsense/splice-site, F290I, F290C, V296M, P307S, P307R, R326H, R326L, R326C, R326S, G332R, G332E, V339A, V339M, V339G, L344Q, L344P, R366P, R366H, R366G, A368T, A368V, R379H, R379C, I407T, I407N, I407S, F414L, F414S, F414C, A462V, K468R, C497G, C497Y, Q527H, Q527P, Y533C, S535F, C537G, C537R, G543A, G543S, G543C, L547H, L547P, L547F, M548I, M548K, G550R, W581R, W581G, W581C, R604Q, R604W, R635W, R635Q, S638F, G646V, G646E, L653W, L653F, I655N, V657A, V657M, R659H, Y660C, V665G, V665L, M674V, R676W, R676Q, G685R, G685E, G685A, D686Y, D686G, R688H, G699R, G699S, G699D, P700L, P700S, P700R, P700Q, P700T, P700A, D702N, D702Y, V704M, V704G, I705F, I705T, I705S, I705N, G707D, G707V, C710S, C710Y, S714C, V716D, V716F, V716I, N717S, N717I, P718L, R720H, R720G, K721R, K721T, Y724C, R729Q, R729W, R729G, F731C, F731L, F731Y, F731I, F732del, F732C, F732S, F732L, E733G, E733A, F734L, F734C, Y735C, Y735N, Y735S, R736H, R736C, R736P, L737H, L737V, L737F, L737R, A741V, P742P, P743R, P743L, R749C, R749L, R749H, R749G, F751L, F751C, F752del, F752C, F752L, F752I, F752V, W753G, W753C, W753R, L754P, L754R, L754H, F755S, F755I, F755L, M761I, M761V, G762C, V763I, S770L, S770W, S770P, R771Q, F772I, F772V, L773R, L773V, E774K, E774D, E774G, I780T, D781G, R792H, W795C, W795L, G796D, G796V, N797Y, N797H, N797S, P799S, P799R, P799H, R803S, R803W, P804L, P804S, K826R, S828N, K829R, T835M, N838D, K841Q, Q842E, P849L, D857N, W860R, E863D, F868S, G869S, G869V, M880V, S881R, S881I, R882H, R882P, R882C, R882G, A884P, A884V, Q886R, L889P, L889R, G890D, G890R, G890S, V895M, P896L, V897G, V897D, R899L, R899H, R899C, L901R, L901H, P904L, F909C, P904Q, A910P, C911R, C911Y	NM_022552
EED	Frameshift/nonsense/splice-site, L240Q, I363M	NM_003797
EP300	Frameshift/nonsense/splice_site, VF1148_1149del, D1399N, D1399Y, P1452L, Y1467N, Y1467H, Y1467C, R1627W, A1629V	NM_001429
ETNK1	N244S, N244T, N244K	NM_018638
ETV6	Frameshift/nonsense/splice-site	NM_001987
EZH2	Frameshift/nonsense/splice-site, Q62R, N102S, F145S, F145C, F145Y, F145L, G159R, E164D, R202Q, K238E, E244K, R283Q, H292R, P488S, R497Q, R561H, T568I, K629E, Y641N, Y641H, Y641S, Y641C, Y641F, D659Y, D659G, V674M, A677G, A677V, R679C, R679H, R685C, R685H, A687V, N688I, N688K, H689Y, S690P, I708V, I708T, I708M, E720K, E740K	NM_001203247
FLT3	V579A, V592A, V592I, F594L, FY590-591GD, D835Y, D835H, D835E, del835	NM_004119
GATA1	Frameshift/nonsense/splice-site	NM_002049
GATA2	Frameshift/nonsense/splice-site, R293Q, N317H, A318T, A318V, A318G, G320D, L321P, L321F, L321V, Q328P, R330Q, R361L, L359V, A372T, R384G, R384K	NM_001145661
GATA3	Frameshift/nonsense/splice-site ZNF domain, R276W, R276Q, N286T, L348V,	NM_001002295
GNA13	I34T, G57S, S62F, M68K, Q134R, Y145F, L152F, E167D, Q169H, R264H, E273K, V322G, V362G, L371F	NM_006572
GNAS	R201(844)S, R201(844)C, R201(844)H, R201(844)L, Q227(870)K, Q227(870)R, Q227(870)L, Q227(870)H, R374(1017)C	NM_016592
GNB1	K57N, K57M, K57E, K57T, I80T, I80N	NM_002074
IDH1	R132C, R132G, R132H, R132L, R132P, R132V, V178I	NM_005896
IDH2	R140W, R140Q, R140L, R140G, R172W, R172G, R172K, R172T, R172M, R172N, R172S	NM_002168
IKZF1	Frameshift/nonsense	NM_006060
IKZF2	Frameshift/nonsense	NM_016260
IKZF3	Frameshift/nonsense	NM_012481
JAK1	T478A, T478S, V623A, A634D, L653F, R724H, R724Q, R724P, T782M, L783F	NM_002227
JAK2	N533D, N533Y, N533S, H538R, K539E, K539L, I540T, I540V, V617F, R683S, R683G, del/ins537-539L, del/ins538-539L, del/ins540-543MK, del/ins540-544MK, del/ins541-543K, del542-543, del543-544, ins11546-547	NM_004972
JAK3	M511T, M511I, A572V, A572T, A573V, R657Q, V715I, V715A	NM_000215

KDM6A Frameshift/nonsense/splice-site, del419 NM_021140
KIT ins503, V559A, V559D, V559G, V559I, V560D, V560A, V560G, V560E, del560, E561K, del579, P627L, P627T, R634W, K642E, K642Q, V654A, V654E, H697Y, H697D, E761D, K807R, D816H, D816Y, D816F, D816I, D816V, D816H, del551-559 NM_000222
KRAS G12D, G12A, G12E, G12V, G13D, G13C, G13Y, G13F, G13R, G13A, G13V, G13E, V14I, T58I, G60D, G60A, G60V, Q61K, Q61E, Q61P, Q61R, Q61L, Q61H, K117E, K117N, A146T, A146P, A146V NM_033360
LUC7L2 Frameshift/nonsense/splice-site NM_016019
MLL Frameshift/nonsense NM_005933
MLL2 Frameshift/nonsense NM_003482
MPL S505G, S505N, S505C, L510P, del513, W515A, W515R, W515K, W515S, W515L, A519T, A519V, Y591D, W515-518KT NM_005373
NF1 Frameshift/nonsense NM_000267
NPM1 Frameshift p.W288fs (insertion at c.859_860, 860_861, 862_863, 863_864) NM_002520
NRAS G12S, G12R, G12C, G12N, G12P, G12Y, G12D, G12A, G12V, G12E, G13S, G13R, G13C, G13N, G13P, G13Y, G13D, G13A, G13V, G13E, G60E, G60R, Q61R, Q61L, Q61K, Q61P, Q61H, Q61Q NM_002524
PDS5B Frameshift/nonsense/splice-site, R1292Q NM_015032
PDSS2 Frameshift/nonsense NM_020381
PHF6 Frameshift/nonsense/splice-site, A40D, M125I, S246Y, F263L, R274Q, C297Y, H302Y, H329L NM_001015877
PHIP Frameshift/nonsense/splice-site NM_017934
PPM1D Frameshift/nonsense, exon 5 or 6 NM_003620
PRPF40B Frameshift/nonsense/splice-site, P15H, M58I, P405L, P562S, NM_001031698
PRPF8 M1307I, C1594W, D1598Y, D1598N, D1598V (ADD MORE VARS) NM_006445
PTEN Frameshift/nonsense/splice-site, D24G, R47G, F56V, L57W, H61R, K66N, Y68H, C71Y, F81C, Y88C, D92G, D92V, D92E, H93Y, H93D, H93Q, N94I, P95L, I101T, C105F, C105S, D107Y, L112V, H123Y, C124R, C124S, K125E, A126D, K128N, R130G, R130Q, R130L, G132D, I135V, I135K, C136R, C136F, K144Q, A151T, D153Y, D153N, Y155H, Y155C, R159K, R159S, R161K, R161I, G165R, G165E, S170N, S170I, R173C, Y174D, Y177C, H196Y, R234W, G251C, D252Y, F271S, D326G NM_000314
PTPN11 G60V, G60R, G60A, D61Y, D61V, D61G, Y63C, E69K, E69G, E69D, E69Q, F71L, F71K, A72T, A72V, A72D, T73I, E76K, E76Q, E76M, E76A, E76G, E139G, E139D, N308D, N308T, N339S, P491L, S502P, S502A, S502L, G503V, G503G, G503A, G503E, Q506P, T507A, T507K NM_002834
RAD21 Frameshift/nonsense/splice-site, R65Q, H208R, Q474R NM_006265
RUNX1 Frameshift/nonsense/splice-site, S73F, H78Q, H78L, R80C, R80P, R80H, L85Q, P86L, P86H, S114L, D133Y, L134P, R135G, R135K, R135S, R139Q, R142S, A165V, R174Q, R177L, R177Q, A224T, D171G, D171V, D171N, R205W, R223C NM_001001890
SETBP1 D868N, D868T, S869N, G870S, I871T, D880N, D880Q NM_015559
SETD2 Frameshift/nonsense, V1190M NM_014159
SETDB1 Frameshift/nonsense, K715E NM_001145415
SF1 Frameshift/nonsense/splice-site, T454M, Y476C, A508G NM_004630
SF3A1 Frameshift/nonsense/splice-site, A57S, M117I, K166T, Y271C NM_005877
SF3B1 G347V, R387W, R387Q, E592K, E622D, Y623C, R625L, R625C, R625G, H662Q, H662D, T663I, K666N, K666T, K666E, K666R, K700E, V701F, A708T, G740R, G740E, A744P, D781G, E783K, R831Q, L833F, E862K, R957Q NM_012433
SFRS2 Y44H, P95H, P95L, P95T, P95R, P95A, P107H, P95fs NM_003016
SMC1A K190T, R586W, M689V, R807H, R1090H, R1090C NM_006306
SMC3 Frameshift/nonsense, R155I, Q367E, D392V, K571R, R661P, G662C NM_005445
STAG1 Frameshift/nonsense/splice-site, H1085Y NM_005862
STAG2 Frameshift/nonsense/splice-site NM_006603
SUZ12 Frameshift/nonsense NM_015355
TET2 Frameshift/nonsense/splice-site, missense mutations in catalytic domains (p.1104-1481 and 1843-2002) NM_001127208
TP53 Frameshift/nonsense/splice-site, S46F, G105C, G105R, G105D, G108S, G108C, R110L, R110C, T118A, T118R, T118I, S127F, S127Y, L130V, L130F, K132Q, K132E, K132W, K132R, K132M, K132N, F134V. F134L, F134S, C135W, C135S, C135F, C135G, C135Y, Q136K, Q136E, Q136P, Q136R, Q136L, Q136H, A138P, A138V, A138A, A138T, T140I, C141R, C141G, C141A, C141Y, C141S, C141F, C141W, V143M, V143A, V143E, L145Q, W146C, W146L, L145R, V147G, P151T, P151A, P151S, P151H, P151R, P152S, P152R, P152L, T155P, T155A, V157F, R158H, R158L, A159V, A159P, A159S, A159D, A161T, A161D, Y163N, Y163H, Y163D, Y163S, Y163C, K164E, K164M, K164N, K164P, H168Y, H168P, H168R, H168L, H168Q, M169I, M169T, M169V, E171K, E171Q, E171G, E171A, E171V, E171D, V172D, V173M, V173L, V173G, R174W, R175G, R175C, R175H, C176R, C176G, C176Y, C176F, C176S, P177R, P177R, P177L, H178D, H178P, H178Q, H179Y, H179R, H179Q, R181C, R181Y, D186G, G187S, P190L, P190T, H193N, H193P, H193L, H193R, L194F, L194R, I195F, I195N, I195T, R196P, V197L, G199V, Y205N, Y205C, Y205H, D208V, R213Q, R213P, R213L, R213Q, H214D, H214R, S215G, S215I, S215R, V216M, V217G, Y220N, Y220H, Y220S, Y220C, E224D, I232F, I232N, I232T, I232S, Y234N, Y234H, Y234S, Y234C, Y236N, Y236H, Y236C, M237V, M237K, M237I, C238R, C238G, C238Y, C238W, N239T, N239S, S241Y, S241C, S241F, C242G, C242Y, C242S, C242F, G244S, G244C, G244D, G245S, G245R, G245C, G245D, G245A, G245V, G245S, M246V, M246K, M246R, M246I, N247I, R248W, R248G, R248Q, R249G, R249W, R249T, R249M, P250L, I251N, L252P, I254S, I255F, I255N, I255S, L257Q, L257P, E258K, E258Q, D259Y, S261T, G262D, G262V, L265P, G266R, G266E, G266V, R267W, R267Q, R267P, E271K, V272M, V272L, R273S, R273G, R273C, R273H, R273P,

R273L, V274F, V274D, V274A, V274G, V274L, C275Y, C275S, C275F, A276P, C277F, C277Y, P278T, P278A, P278S, P278H, P278R, P278L, G279E, R280G, R280K, R280T, R280I, R280S, D281N, D281H, D281Y, D281G, D281E, R282G, R282W, R282Q, R282P, E285K, E285V, E286G, E286V, E286K, K320N, L330R, G334V, R337C, R337L, A347T, L348F, T377P NM_001126112

U2AF1 D14G, S34F, S34Y, R35L, R156H, R156Q, Q157R, Q157P NM_006758

U2AF2 R18W, Q143L, M144I, L187V, Q190L NM_007279

WT1 Frameshift/nonsense/splice-site NM_024426

ZRSR2 Frameshift/nonsense, R126P, E133G, C181F, H191Y, I202N, F239V, F239Y, N261Y, C280R, C302R, C326R, H330R, N382K NM_005089

Table S4. The multivariable logistic regression models testing a potential effect of the coverage in the matched sub cohorts. Models were run using the glm function with the setting “family="binomial" in R.

model	trait	coefficient	p-value
**1: CHIP carrier (0/1) ~ cohort (0/1)**	**intercept**	**-3.5492**	**< 2e-16**
	**cohort (0/1)**	**0.9342**	**0.00452**
2: CHIP carrier (0/1) ~ cohort (0/1) + Total Coverage (DNMT3A + TET2 + JAK2 + ASXL1)<br><br>After removing the ‘total coverage’ the model converged to the model 1	intercept	-0.731221	7.74e-15
	cohort (0/1)	0.731221	0.0622
	total coverage	0.002350	0.3188
3: CHIP carrier (0/1) ~ cohort (0/1) + coverage of DNMT3A + coverage of TET2 + coverage of JAK2 + coverage of ASXL1<br><br>After step by step removing of the least significant variables, the model converged to the model 1	intercept	-4.066032	4.48e-14
	cohort (0/1)	0.733876	0.0866
	coverage of DNMT3A	-0.007368	0.6921
	coverage of TET2	-0.011276	0.4340
	coverage of JAK2	0.004443	0.7310
	coverage of ASXL1	0.025918	0.3376
